# Pattern of callose deposition during the course of meiotic diplospory in *Chondrilla juncea* (Asteraceae, Cichorioideae)

**DOI:** 10.1007/s00709-016-1039-y

**Published:** 2016-11-05

**Authors:** Krystyna Musiał, Maria Kościńska-Pająk

**Affiliations:** Department of Plant Cytology and Embryology, Institute of Botany, Jagiellonian University, Gronostajowa 9, 30-387 Cracow, Poland

**Keywords:** Apomixis, Callose, Megasporogenesis, Ovule, *Chondrilla*, Rush skeletonweed

## Abstract

Total absence of callose in the ovules of diplosporous species has been previously suggested. This paper is the first description of callose events in the ovules of *Chondrilla juncea*, which exhibits meiotic diplospory of the *Taraxacum* type. We found the presence of callose in the megasporocyte wall and stated that the pattern of callose deposition is dynamically changing during megasporogenesis. At the premeiotic stage, no callose was observed in the ovules. Callose appeared at the micropylar pole of the cell entering prophase of the first meioticdivision restitution but did not surround the megasporocyte. After the formation of a restitution nucleus, a conspicuous callose micropylar cap and dispersed deposits of callose were detected in the megasporocyte wall. During the formation of a diplodyad, the micropylar callose cap decreased and the walls of a newly formed megaspores showed scattered distribution of callose. Within the older diplodyad, callose was mainly accumulated in the wall between megaspores, as well as in the wall of the micropylar cell; however, a dotted fluorescence of callose was also visible in the wall of the chalazal megaspore. Gradual degradation of callose in the wall of the chalazal cell and intense callose accumulation in the wall of the micropylar cell were related to the selection of the functional megaspore. Thus, our findings may suggest that callose fulfills a similar role both during megasporogenesis in sexual angiosperms and in the course of meiotic diplospory in apomicts and seems to form a regulatory interface between reproductive and somatic cells.

## Introduction

Callose, a β-1,3-linked homopolymer of glucose containing some β-1,6 branches, may be considered as a histological marker for a preliminary identification of the reproduction mode in angiosperms. In the majority of sexually reproducing flowering plants, the isolation of the spore mother cell and the tetrad by callose walls is a striking feature of both micro- and megasporogenesis (Rodkiewicz 1970; Bhandari 1984; Bouman 1984; Lersten 2004). Callose functions as a marker to distinguish the reproductive cells from other ovule tissue in plants with mono- and bisporic patterns of megasporogenesis (Rodkiewicz 1970; Russell 1979; Tucker and Koltunow 2014). Moreover, uneven callose deposition and degradation during female meiosis is one of the factors involved in the designation of abortive megaspores and thereby crucial in the selection of a functional megaspore within a tetrad regulating the selection of a functional megaspore within a tetrad and appears to be linked to the programmed cell death of three supernumerary megaspores (Webb and Gunning 1990; Papini et al. 2011). Contrary to sexual reproduction, the pattern of callose deposition is altered in the ovules of apomicts (Drews and Koltunow 2011; Galla et al. 2011; Musiał et al. 2015). As a rule, callose is absent in the walls of the cells that initiate diplospory and apospory, which may suggest that these cells do not share identity with functional megaspore mother cells (Tucker et al. 2001; Bicknell and Koltunow 2004). However, it should be noted that research data on the callose accumulation and degradation in the ovules of apomicts, especially diplosporous species, are not unambiguous and still remain poorly documented. Total absence of callose or an incomplete callose wall in the megasporocyte was essentially observed in the species exhibiting mitotic diplospory (*Antennaria* type, in which meiosis is omitted), for example in the grasses *Elymus rectisetus* (Carman et al. 1991), *Poa nemoralis* and *Poa palustris* (Naumova et al. 1993, 1999), *Tripsacum* species (Leblanc et al. 1993, 1995), and *Eragrostis curvula* (Peel et al. 1997). It has been postulated that the lack of callose during megasporogenesis may be also a characteristic of the meiotic diplospory (Carman et al. 1991; Peel et al. 1997). However, callose walls around the megaspore mother cells were found in *Paspalum minus*, which showed meiotic diplospory of the *Taraxacum* type that involves first division restitution and normal second meiotic division (Bonilla and Quarin 1997). Recently, callose deposition has also been documented during meiotic diplospory in the ovules of a triploid dandelion *Taraxacum atricapillum* (Musiał et al. 2015). As there is a close phylogenetic relationship between *Taraxacum* and *Chondrilla*, as well as a similarity of apomixis mechanisms in both of these genera (van Dijk 2003), in the present research, we have undertaken an analysis of callose location in the young ovules of diplosporous species *Chondrilla juncea* L.

The genus *Chondrilla* L. represents the Asteraceae family and belongs to the Cichorioideae subfamily, the tribe Cichorieae, and the subtribe Chondrillinae (Koopman et al. 1998; Anderberg et al. 2007; Kilian et al. 2009). It includes a polyploid complex comprising both amphimictic diploids (2*n* = 2 × = 10) and agamospermous polyploid species, mainly triploids (2*n* = 3 × = 15) and tetraploids (2*n* = 4 × = 20) (Poddubnaja-Arnoldi 1933; Bergman 1950, 1952a, b; Kościńska-Pająk 1996; Van Dijk 2003). There are about 30 species of *Chondrilla* worldwide which are biennial or perennial hemicryptophytes (Iljin 1930; McVean 1966; Kościńska-Pająk 1996; van Dijk 2003). The genus *Chondrilla* is native to Eurasia and North Africa (Iljin 1930; van Dijk 2003); however, certain taxa have been introduced in Australia, and North and South America, where especially *C. juncea* (rush skeletonweed) became a rapidly spreading noxious invasive weed in cereal cultivations (McVean 1966; Gaskin et al. 2013). So far, *C. juncea* is the most studied taxon within the genus. Extensive research is designed to improve the understanding of its invasion and to introduce an effective program of biological control. *C. juncea* is a triploid perennial herb that reproduces clonally via autonomous gametophytic apomixis, and hence, the formation of viable seeds is completely independent of the male gametophyte (Iljin 1930; Poddubnaja-Arnoldi 1933; Jankun et al. 1996). Research on the mode of reproduction showed the occurrence of meiotic diplospory of the *Taraxacum* type, parthenogenesis, and autonomous endosperm formation in this species (Rosenberg 1912; Poddubnaja-Arnoldi 1933; Battaglia 1949; Bergman 1950; Cuthbertson 1974; Kościńska-Pająk 1996, 2006). The subsequent embryological examinations, conducted on the specimens of *C. juncea* from natural habitats in Poland, were also devoted to a study of the male gametophyte development and the pattern of cytoskeletal organization during microsporogenesis, as well as the microtubule configuration in the cells of a diplosporous female gametophyte (Kościńska-Pająk 2000, 2006; Kościńska-Pająk and Bednara 2003, 2006).

Up to now, callose events in the ovules have never been studied in detail in the genus *Chondrilla*, though preliminary observations of *C. juncea* young ovules revealed the presence of a thick callose wall between the unreduced megaspores within the diplodyad (Kościńska-Pająk 2006). The aim of the present investigation was to examine the pattern of callose deposition during meiotic diplospory of the *Taraxacum* type in the ovules of *C. juncea*.

## Materials and methods

### Plant material

Mature seeds of *C. juncea* were sampled from plants within a natural population in Poland, locality Jany (51° 58′ 26″ N, 15° 36 25″ E). Then, the plants obtained from the seeds were grown on an experimental field in Modlnica near Cracow (50° 07′ 45″ N, 19° 52′ 01″ E). From the cultured specimens, young capitula were collected and fixed in glacial acetic acid: 96 % ethanol (1:3, *v*/*v*) for at least 24 h. The fixed plant material was transferred to 70 % ethanol and stored at 4 °C.

### Tissue clearing technique

Individual flowers were isolated from fixed inflorescences and dehydrated for 30 min in a graded ethanol series (70 to 100 %). Then they were cleared in methyl salicylate (Sigma-Aldrich) according to a procedure earlier described by Musiał et al. (2012) with some modifications. Dehydrated flowers were incubated in absolute ethanol/methyl salicylate solutions (3:1, 1:1, and 1:3, *v*/*v*) and in two changes of pure methyl salicylate (1 h per step). Cleared samples were placed in a drop of pure methyl salicylate on a Raj slide (Herr 2000) and examined using a Nikon Eclipse 80i microscope fitted with Nomarski’s Interference Contrast (DIC optics). A total of 67 ovules were analyzed.

### Detection of callose

Decolorized aniline blue (DAB; 0.1 % *w*/*v*) was used to detect the presence of callose (Martin 1959). Individual flowers were dissected from fixed capitula and transferred to 80 % ethanol for 30 min. Then they were softened in 1 N NaOH for 4 h at 37 °C, and after three washes with distilled water and one with 0.1 M K_3_PO_4_, the softened samples were stained overnight in 0.1 % DAB in 0.1 M K_3_PO_4_ at room temperature. After washing with 0.1 M K_3_PO_4_, flowers were placed into a drop of 0.1 M K_3_PO_4_/glycerol (1:1, *v*/*v*) on a microscope slide and ovules were dissected under a stereomicroscope. After ovule isolation, samples were gently squashed under a cover slip and observed under UV light using a Nikon Eclipse E400 microscope with an Epi-Fl Filter Block N UV-2A consisting of excitation filter EX330–380, dichroic mirror DM400, and barrier filter BA420. A total of 146 ovules were analyzed.

## Results

### Early ovule development and megasporogenesis

**Fig. 1:**
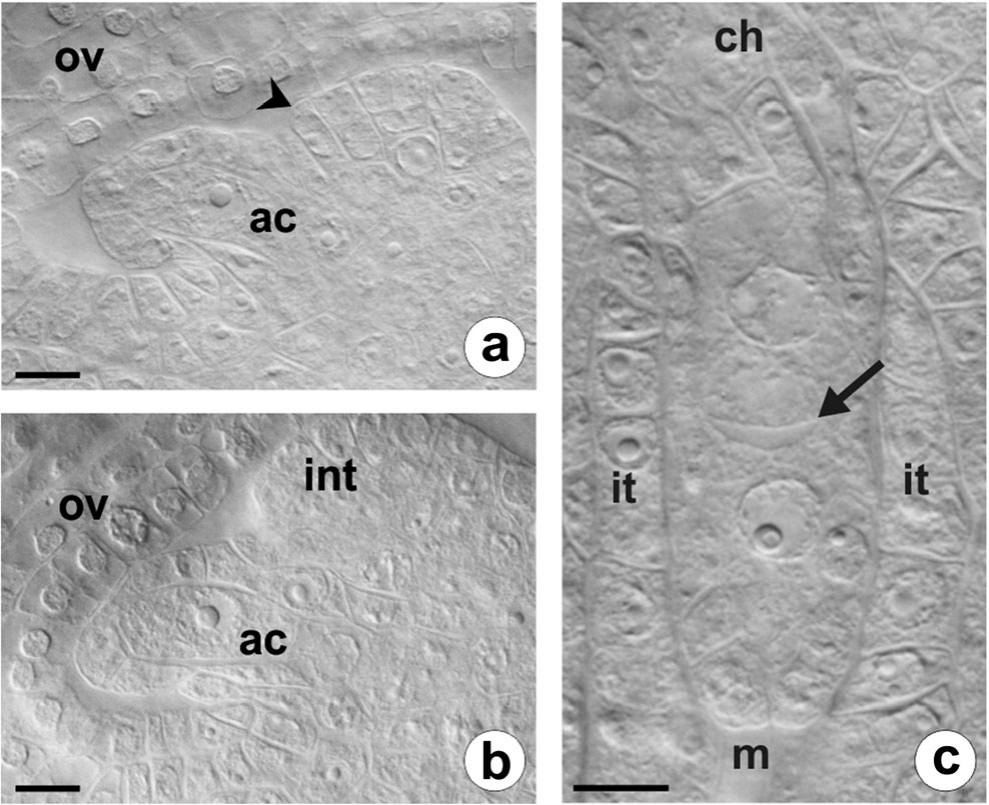
Early stages of the ovule formation in *C. juncea*. Images were obtained from cleared flowers using DIC optics. **a**, **b** Unilocular ovary containing ovule primordium with a single archesporial cell differentiated in the hypodermal part of the nucellus and a visible developing integument. *ac* archesporial cell, *int* integument, *ov* ovary wall; *arrowhead* indicates region of integument initiation. **c** Dyad of unreduced megaspore cells surrounded by a layer of integumentary tapetum. *Arrow* points to thick transversal wall between megaspores. *ch* chalazal pole, *it* integumentary tapetum, *m* micropylar pole. *Scale bars* = 10 μm

**Fig. 2:**
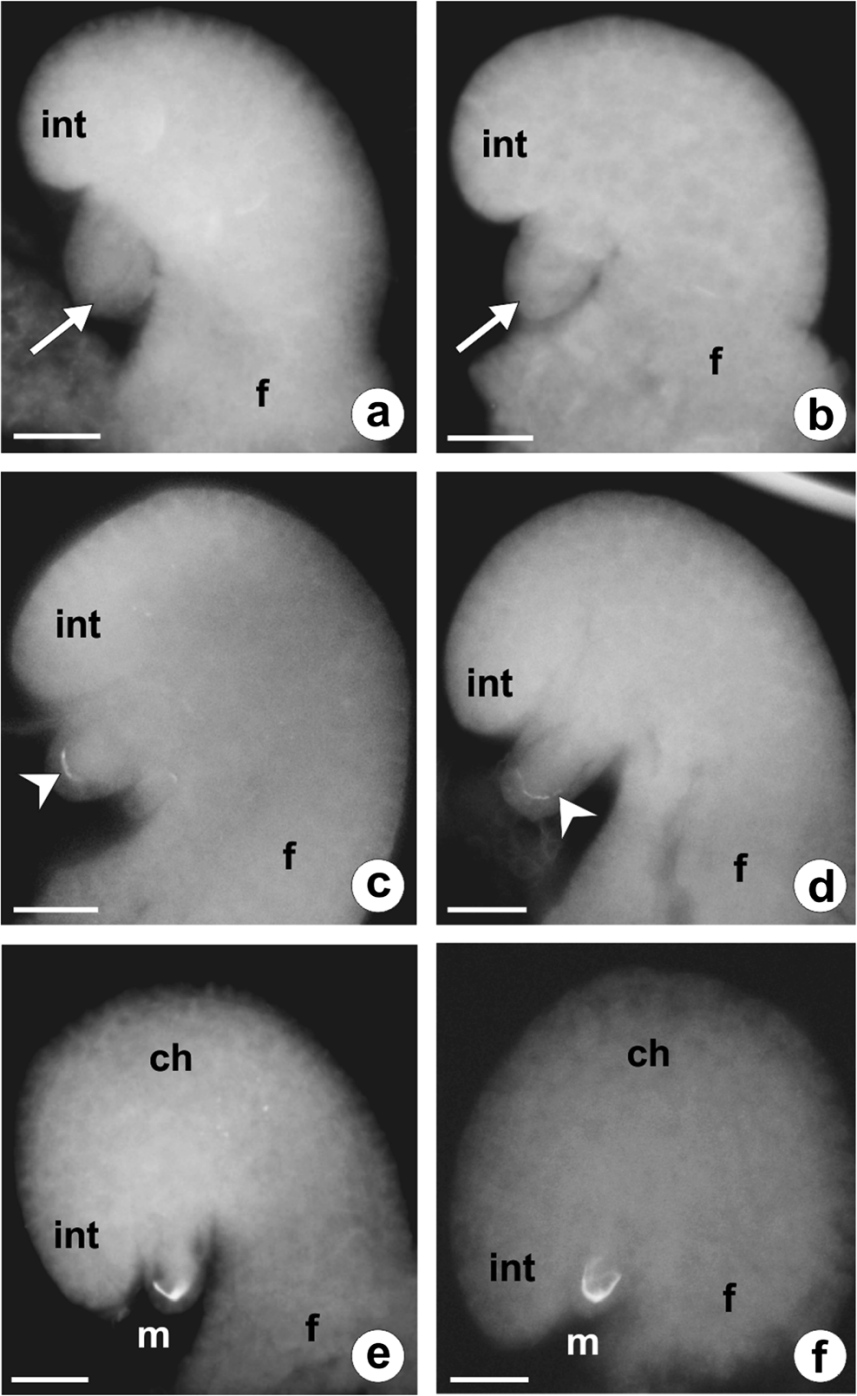
Callose localization in the ovules of *C. juncea* after staining with DAB. **a**, **b** Early developmental stages of an anatropous, unitegmic, and tenuinucellate ovule. Callose is absent in the walls of both somatic and archesporial cells (*arrow*). **c**, **d** Megaspore mother cell entering prophase of the first division restitution. Callose appears at the megasporocyte micropylar pole as a fine arc-shaped line (*arrowhead*). **e**, **f** Late prophase I in the megaspore mother cell. Callose fluorescence is visible only at the megasporocyte micropylar pole. *ch* chalazal pole, *f* funicle, *int* integument, *m* micropylar pole. *Scale bars* = 10 μm

A homogamous capitula of *C. juncea* comprise from 9 to 11 yellow ligulate florets, which are hermaphroditic and have a bicarpellate gynoecium with an inferior, unilocular ovary and five stamens with connate anthers forming a tube around the pistil style. In *C. juncea*, as in other members of Asteraceae, mature ovules are anatropous, tenuinucellate, and unitegmic. In the examined florets, one ovule primordium emerged from the placental tissue at the base of the young ovary, and, just after ovule initiation, a single archesporial cell differentiated in the subepidermal zone of the nucellus apex (Fig. 1a, b). This distinctly enlarged cell had a dense cytoplasm and a centrally located prominent nucleus with a large nucleolus (Fig. 1a). The archesporial cell extended along the micropylar-chalazal axis, and simultaneously, a single integument began to develop at the base of the nucellus (Figs. 1a, b and 2a, b). During further ovule development, the multilayer integument gradually covered the nucellus leaving a small apical opening—the micropyle, and because of curvature of the funiculus, the anatropous orientation of the ovule was established (Fig. 2a–e). The archesporial cell functioned directly as the megaspore mother cell (MMC) and entered an asyndetic meiotic prophase. A disturbed first meiotic division led to the formation of a restitution nucleus with an unreduced chromosome number, whereas the second meiotic division proceeded without irregularities and resulted in a dyad of unreduced megaspores (Fig. 1c). After the completion of meiosis, the inner epidermis of the integument began to develop in the integumentary tapetum surrounding a single layer of nucellar cells adjacent to the diplodyad (Fig. 1c). Then, the micropylar cell of the diplodyad gradually degenerated while the chalazal one survived and became the functional megaspore (FM), which gave rise to an unreduced female gametophyte by three successive mitoses.

### Callose localization in young ovules

**Fig. 3:**
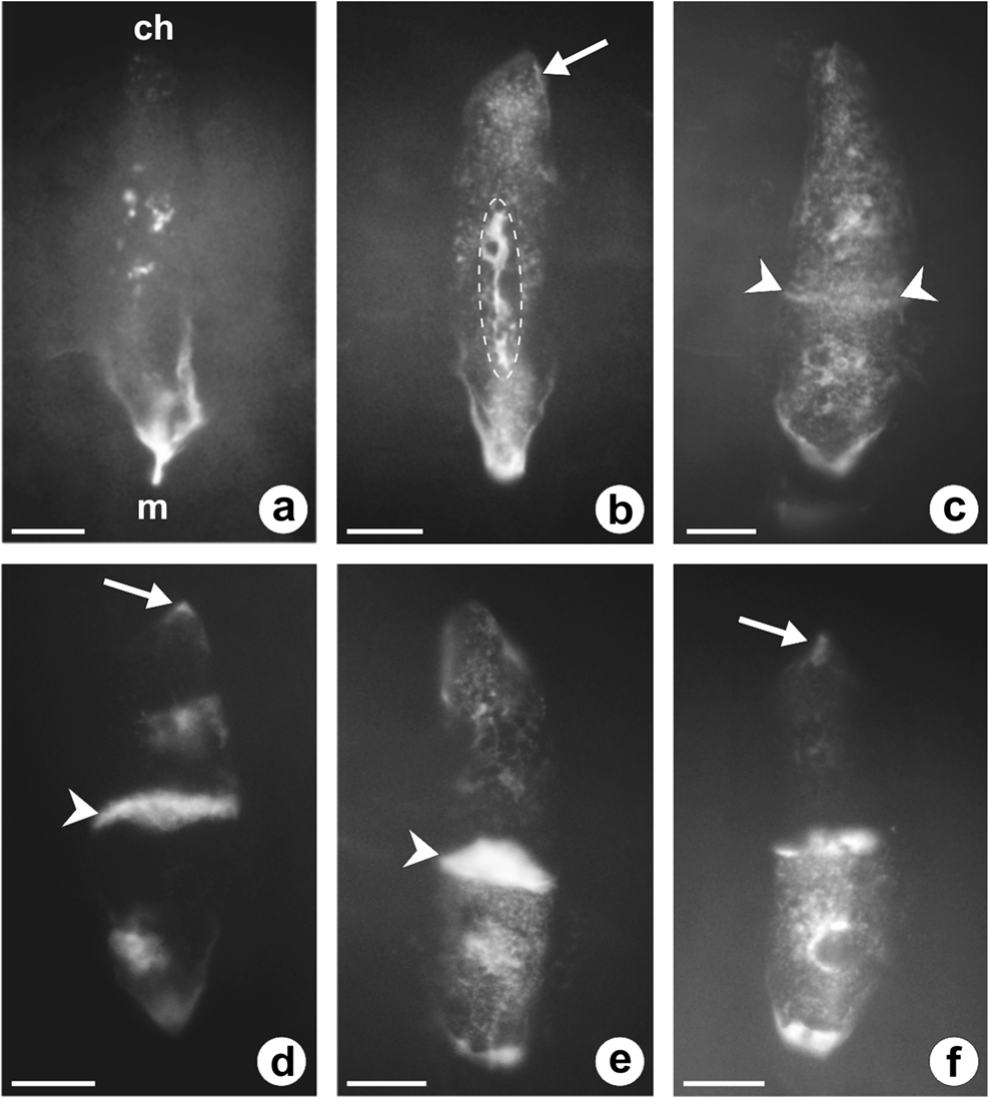
Callose localization during diplodyad formation in the ovules of *C. juncea* after staining with DAB. **a**, **b** Megaspore mother cell with a restitution nucleus. Strong fluorescence of the micropylar cap of callose and slight callose deposits at the chalazal pole (*arrow*) are visible. **c** Megaspore mother cell just after the second meiotic division. Note the decrease of micropylar callose cap fluorescence and its appearance in the plate cell (*arrowheads*). **d** Young diplodyad. Callose is mainly accumulated in the transversal wall (*arrowhead*); dotted fluorescence is visible in the wall of the chalazal megaspore (*arrow*). **e**, **f** Formation of the functional megaspore. Gradual degradation of callose in the wall of the chalazal cell within the diplodyad and an intensive callose accumulation in the wall of the micropylar cell, as well as in the transversal wall between megaspores (*arrowhead*); *arrow* indicates a remaining small callose deposit at the chalazal pole of the functional megaspore. *ch* chalazal pole, *m* micropylar pole. *Scale bars* = 10 μm

At the premeiotic stage, callose was absent in the walls of the ovule somatic cells, as well as in the wall of the archesporial cell (Fig. 2a, b). In the slightly older ovules of *C. juncea* under examination, the presence of callose was limited to the walls of the germline cells. The appearance of callose was observed just after the beginning of the first meiotic division restitution, and the amount of deposited callose, as well as the pattern of its distribution, changed dynamically in the course of the meiotic diplospory and during the FM development. Callose biosynthesis was initiated at the stage of prophase I, and fluorescence of the aniline blue-stained callose deposits was noticeable at the micropylar apex of the MMC wall in the form of a fine arc-shaped line (Fig. 2c). Then, deposition of callose progressed slightly towards the chalazal pole of the MMC (Fig. 2d); however, the wall comprising callose did not extend to the entire megasporocyte (Figs. 2e, f and 3a). Moreover, callose was not evenly deposited in the wall of MMC and its greatest accumulation was observed at the top of the megasporocyte micropylar pole (Figs. 2e, f and 3a, b). Strong fluorescence of the micropylar cap of callose was observed until the end of the first meiotic division restitution (Fig. 3a, b). After the formation of restitution nucleus, a very slight callose deposition was also found at the top of the MMC chalazal pole (Fig. 3b). In addition, especially in the middle part of the lateral wall and in the chalazal region of the MMC, scattered and dotted callose distribution was observed (Fig. 3b). Usually, dispersed deposits of callose were still visible in the walls of newly formed megaspores at the end of the second meiotic division (Fig. 3c); however, in some diplodyads, the lateral walls of megaspores did not exhibit such a specific callose accumulation (Fig. 3d). During cytokinesis, callose appeared in the cell plate, while an intense fluorescence of the callose micropylar cap significantly decreased but did not disappear completely (Fig. 3c). In the diplodyad, a newly formed transverse wall between the megaspores showed enhanced callose deposition and over time, these callose deposits took the shape of a thick disk exhibiting a very strong fluorescence (Fig. 3d, e). While the chalazal megaspore of the diplodyad developed into the FM, the intensity of callose fluorescence gradually decreased in this cell (Fig. 3e, f). Finally, the lateral wall of the FM was devoid of callose and showed no fluorescence, and only a very small amount of callose persisted at the top of its chalazal pole (Fig. 3f). At the same time, the wall of the micropylar cell of the diplodyad displayed stronger fluorescence due to an increased accumulation of callose, which again formed a distinctive cap at the top of the megaspore micropylar pole (Fig. 3f).

## Discussion

It was suggested previously that the absence of callose during megasporogenesis may be common among diplosporous apomicts (Carman et al. 1991; Peel et al. 1997). However, the results of our study do not confirm this hypothesis and distinctly indicate the presence of callose in the young ovules of some diplosporous species exhibiting meiotic diplospory of the *Taraxacum* type. *C. juncea* is the second apomictic species in which we described the pattern of callose accumulation in the wall of a cell undergoing meiotic diplospory. Earlier, we documented the details of callose deposition in the walls of MMC and during the FM selection in the ovules of a diplosporous dandelion *T. atricapillum* (Musiał et al. 2015). In the ovules of these two apomictic species, as in sexual reproducing angiosperms, this polysaccharide appears as a transient wall component only of the cells involved in the reproductive process. Usually, callose deposition patterns vary between species (Rodkiewicz 1970); however, ovules of apomicts *C. juncea* and *T. atricapillum* show a similar pattern of its accumulation, and during the first meiotic division restitution, abundant callose deposits were detected on the micropylar pole of the MMC. In the case of *Taraxacum*, there were differences in the deposition of callose between apomictic and sexual species. The monopolar pattern of its deposition in the wall of the MMC was observed only in the ovules of an apomictic dandelion, while in a sexual diploid *Taraxacum linearisquameum*, callose was deposited in a bipolar manner in the wall of the megasporocyte (Musiał et al. 2015). Regarding the genus *Chondrilla*, it would be also interesting to compare callose events in the ovules of apomictic and sexual taxa; unfortunately, now we do not have the specimens of diploid species.

Callose has multiple biological functions and plays an important role in the regulation of intercellular communication during developmental, physiological, and stress response processes in flowering plants; however, the molecular mechanisms involved in its biosynthesis and degradation have not yet been fully elucidated (Verma and Hong 2001; Chen and Kim 2009; Zavaliev et al. 2011; Piršelová and Matušíková 2013). It has been shown that callose deposition is an early marker in somatic embryogenesis and in this case, its possible role is that of isolating an embryogenic cell from the influence of the surrounding cells and interrupting cell–cell communication, which might stimulate the reprogramming of a somatic cell into an embryogenically competent cell and induce somatic embryo development (Dubois et al. 1990, 1991; You et al. 2006). Likewise, temporary callose walls surrounding the zygote and young zygotic embryo may have a function in establishing spatial isolation and may allow for the initiation of genome reprogramming and the first division of the zygote, as well as early embryogenesis (Williams et al. 1984). Distinct callose walls are also found around the initials of nucellar embryos. They also surround young adventitious embryos (Gupta et al. 1996). Thus, in the light of the postulated callose role as a factor isolating cells undergoing an autonomous genetic program that determines especial cell differentiation, the lack of callose in the walls of the aposporous initials in apomicts (Tucker et al. 2001), as well as its absence during tetrasporic megasporogenesis in sexual angiosperms (Rodkiewicz 1970), seems intriguing. Recent studies have confirmed the key role of intercellular signaling between the somatic tissues and the reproductive lineage during both sexual and apomictic female reproductive developments (Armenta-Medina et al. 2011; Bencivenga et al 2011; Grossniklaus 2011; Tucker et al 2012). Currently, it is known that these signaling pathways involve transcriptional regulation by transcription factors and posttranscriptional control mechanisms, and epigenetic regulation via small RNAs, as well as hormonal regulation (for review see Drews and Koltunow 2011; Grimanelli 2012; Rodriguez-Leal and Vielle-Calzada 2012; Barcaccia and Albertini 2013; Schmidt et al. 2014, 2015). During the early ovule development, callose is a wall component only of a differentiating female reproductive cell and seems to form a regulatory interface between reproductive and somatic cells, but it is still unclear whether this polysaccharide functions as a specific semipermeable molecular filter, or rather as a source of oligosaccharide signaling molecules influencing cell differentiation and fate (Tucker and Koltunow 2009, 2014). In sexual species, the pattern of callose deposition during megasporogenesis is also essential to the selection the FM, and callose represents a physical barrier that suppresses nonfunctional megaspores by restricting the flow of nutrients or growth factors (Rodkiewicz 1970; Russell 1979; Webb and Gunning 1990; Papini et al. 2011). Our previous observations of callose events in the ovules of apomict *T. atricapillum* (Musiał et al. 2015) and result of the present analysis of callose deposition in the ovules of *C. juncea* indicate that in diplosporous apomicts exhibiting meiotic diplospory of the *Taraxacum* type, the pattern of callose distribution is also related to the selection of the FM, and disappearance of callose within the diplodyad coincides with the localization of the FM.

In conclusion, the present report documents, for the first time, callose events in the course of meiotic diplospory in *C. juncea*. In the analyzed ovules, we recorded (i) lack of callose in the premeiotic stage; (ii) callose is a marker of the cell entering the first meiotic division restitution; (iii) monopolar callose deposition in the megasporocyte wall; (iv) callose deposition pattern changing dynamically during the diplodyad formation; and (v) callose deposition and dissolution corresponding to the localization of the FM.
